# Toxocariasis in Waste Pickers: A Case Control Seroprevalence Study

**DOI:** 10.1371/journal.pone.0054897

**Published:** 2013-01-22

**Authors:** Cosme Alvarado-Esquivel

**Affiliations:** Department of Infectology, Faculty of Medicine and Nutrition, Juárez University of Durango State, Durango, Mexico; Albert Einstein College of Medicine, United States of America

## Abstract

**Background:**

The epidemiology of *Toxocara* infection in humans in Mexico has been poorly explored. There is a lack of information about *Toxocara* infection in waste pickers.

**Aims:**

Determine the seroepidemiology of *Toxocara* infection in waste pickers.

**Methods:**

Through a case control study design, the presence of anti-*Toxocara* IgG antibodies was determined in 90 waste pickers and 90 age- and gender-matched controls using an enzyme-linked immunoassay. Associations of *Toxocara* exposure with socio-demographic, work, clinical, and behavioral data of the waste pickers were also evaluated.

**Results:**

The seroprevalence of anti-*Toxocara* IgG antibodies was significantly higher in waste pickers (12/90: 13%) than in control subjects (1/90: 1%) (OR  = 14; 95% CI: 2–288). The seroprevalence was not influenced by socio-demographic or work characteristics. In contrast, increased seroprevalence was found in waste pickers suffering from gastritis, and reflex and visual impairments. Multivariate analysis showed that *Toxocara* exposure was associated with a low frequency of eating out of home (OR  = 26; 95% CI: 2–363) and negatively associated with consumption of chicken meat (OR  = 0.03; 95% CI: 0.003–0.59). Other behavioral characteristics such as animal contacts or exposure to soil were not associated with *Toxocara* seropositivity.

**Conclusions:**

1) Waste pickers are a risk group for *Toxocara* infection. 2) *Toxocara* is impacting the health of waste pickers. This is the first report of *Toxocara* exposure in waste pickers and of associations of gastritis and reflex impairment with *Toxocara* seropositivity. Results warrant for further research.

## Introduction

Infection with the parasite *Toxocara* is among the most common zoonotic infections worldwide [1], [2]. The *Toxocara* eggs are present in dogs and cat feces and become infectious within weeks after they are deposited in the local environment [3], [4]. When embryonated eggs are accidentally ingested by humans, larvae hatch in the small intestine, penetrate the intestinal wall and migrate, via the bloodstream, to anywhere in the body including liver, lungs, muscles, eye, and central nervous system [2], [5]. Human infection may also occur by ingesting *Toxocara* larvae from undercooked giblets [6]. Most human infections with *Toxocara* are asymptomatic; however, *Toxocara* may lead to serious illness and death [1], [2], [7]. Ocular toxocariasis causes permanent vision loss in many patients [8]. There is poor understanding of the global impact and cost of human toxocariasis [9]. To my knowledge, there is not any report in the medical literature about the epidemiology of *Toxocara* infection in waste pickers. This group of population lives under disadvantaged socioeconomic conditions including poor housing, food, and sanitation, and has very low hygiene practices. In addition, waste pickers have not social security for covering health care services as diagnosis, treatment, and prevention of infectious diseases. This study was aimed to determine the seroprevalence of *Toxocara* infection in waste pickers in Durango, Mexico and to identify their characteristics associated with *Toxocara* seropositivity.

## Methods

Through an age- and gender-matched case-control study using serum samples from recent *Toxoplasma gondii* serosurveys [10], [11], 90 waste pickers and 90 control subjects were compared for the presence of anti-*Toxocara* IgG antibodies. Inclusion criteria for the waste pickers were: 1) waste pickers in the Municipal solid waste transfer station of Durango City, Mexico; 2) aged 14 years and older; 3) any gender; 4) waste picking for at least 3 months; and 5) who accepted to participate in the study. Waste pickers were 14–76 (mean  = 36.0+/−17.1) years old, 34 were males and 56 were females. Control subjects were matched with waste pickers by age and gender and consisted of 34 males and 56 females with miscellaneous occupations other than waste picking including students of public schools, employees, factory workers, housewives, business, and others. The mean age in controls was 35.7±16.8 (range: 18–78) years and comparable with that in waste pickers (*P* = NS).

This study was approved by the Ethical Committee of the Instituto de Seguridad y Servicios Sociales de los Trabajadores del Estado in Durango City. The purpose and procedures of the study were explained to all participants. A written informed consent was obtained from all participants.

The characteristics of the participants were obtained by using a standardized questionnaire. Socio-demographic data including age, gender, birth place, residence, educational level, and socioeconomic level were obtained from all participants. Work characteristics included seniority in the activity, habitual use of safety practices (use of hand gloves and face masks), eating while working, drinking alcohol while waste picking, washing hands before eating, eating from the garbage, and ever had suffered from injuries with sharp material of the garbage. Clinical data explored included the presence of underlying diseases, memory, reflex, hearing, and visual impairments, and history of blood transfusion or transplants. Behavioral data included animal contacts, traveling, meat consumption (pork, beef, goat, mutton, boar, chicken, turkey, rabbit, venison, squirrel, horse, or other), consumption of raw or undercooked meat, unpasteurized milk, dried or cured meat (ham, sausages, salami or chorizo), consumption of unwashed raw vegetables, fruits, or untreated water, frequency of eating out of home (in restaurants or fast food outlets), contact with soil (gardening or agriculture), and types of floors at home from all participants were obtained.

Serum samples were obtained from all participants and kept frozen at −20°C until analyzed. Serum samples were analyzed for anti-*Toxocara* IgG antibodies with a commercially available enzyme immunoassay “*Toxocara*” kit (Diagnostic Automation, Inc. Calabasas, CA, U.S.A.). Absorbance reading equal to or greater than 0.3 OD units was considered positive. All tests were performed following the instructions of the manufacturer.

The statistical analysis was performed with the aid of the software Epi Info version 3.5.3 and SPSS version 15.0. The Pearson's chi-square test and the Fisher exact test (when values were less than 5) were used for comparison of the frequencies among groups. Age in cases and controls was compared with the student *t* test. Bivariate and multivariate analyses were used to assess the association between the characteristics of the waste pickers and *Toxocara* seropositivity. Variables were included in the multivariate analysis if they had a *P* value equal to or less than 0.20 in the bivariate analysis. Odd ratio (OR) and 95% confidence interval (CI) were calculated by multivariate analysis using multiple, unconditional logistic regression. A *P* value less than 0.05 was considered statistically significant.

## Results

The seroprevalence of anti-*Toxocara* IgG antibodies was significantly higher in waste pickers (12/90: 13%) than in control subjects (1/90: 1%) (OR  = 14; 95% CI: 2–288; *P*<0.01). General socio-demographic characteristics of the waste pickers studied are shown in Table 1. *Toxocara* seroprevalence was not influenced by gender, age, residence, educational level, or socioeconomic status of waste pickers.

**Table 1:** Seroprevalence (%) of toxocaral infection in waste pickers relative to bivariate analysis of sociodemographic variables.

Characteristic	No. Of subjects tested^a^	Prevalence	Odds ratio	95% Confidence interval	*P* value
Gender					
Male	34	11.8	1.0		
Female	56	14.3	1.3	0.30–5.47	0.49
Age groups (years)					
30 or less	46	15.2	1.0		
31–50	22	18.2	1.2	0.26–5.61	0.50
>50	21	4.8	0.3	0.01–2.57	0.21
Residence place					
Durango State	88	13.6	1.0		
Other Mexican State	2	0.0	0.0	0.00–29.35	0.75
Residence area					
Urban	83	12.0	1.0		
Suburban or rural	6	16.7	1.5	0.03–15.11	0.55
Educational level					
No education	28	17.9	1.0		
1–6 years	50	10.0	0.5	0.11–2.32	0.25
More than 6 years	12	16.7	0.9	0.10–7.00	0.65
Socioeconomic level					
Low	67	14.9	1.0		
Medium	17	11.8	0.8	0.10–4.37	0.54

a Participants with available data.

None of the work characteristics in waste pickers including seniority in the activity, habitual use of safety practices, eating while working, drinking alcohol while waste picking, washing hands before eating, eating from the garbage, and ever had suffered from injuries with sharp material of the garbage influenced the prevalence of *Toxocara* seropositivity.

With respect to clinical data (Table 2), the prevalence of *Toxocara* seropositivity was significantly (*P*<0.05) higher in waste pickers suffering from gastritis and reflex impairment than those without such clinical features. Waste pickers with visual impairment had a higher (with borderline significance: *P* = 0.05) prevalence of *Toxocara* seropositivity than those without this clinical characteristic. The frequencies of other clinical characteristics including memory and hearing impairments, blood transfusion and transplant history were similar among *Toxocara* positive and *Toxocara* negative waste pickers.

**Table 2:** Seroprevalence (%) of toxocaral infection in waste pickers relative to bivariate analysis of clinical characteristics.

Characteristic	No. Of subjects tested^a^	Prevalence	Odds ratio	95% Confidence interval	*P* value
Health status					
Healthy	78	11.5	1.0		
Ill	10	30.0	3.3	0.55–18.34	0.13
Gastritis					
Yes	3	66.7	15.0	0.93–464.17	0.04
No	85	11.8	1.0		
Memory impairment					
Yes	20	25.0	3.0	0.70–12.69	0.09
No	70	10.0	1.0		
Reflex impairment					
Yes	10	40.0	6.0	1.12–32.36	0.02
No	80	10.0	1.0		
Hearing impairment					
Yes	7	0.0	-	-	0.34
No	82	14.6	1.0		
Visual impairment					
Yes	18	27.8	3.6	0.82–15.45	0.05
No	72	9.7	1.0		
Blood transfusion					
Yes	9	33.3	4.0	0.65–23.31	0.09
No	81	11.1	1.0		
Transplantation					
Yes	2	0	-	-	0.74
No	88	13.6	1.0		

a Participants with available data.

Concerning behavioral characteristics (Table 3), the bivariate analysis showed 7 characteristics with a *P* value equal to or less than 0.20 including: cats in the neighborhood (*P* = 0.07), traveling abroad (*P* = 0.09), consumption of pork (*P* = 0.13), beef (*P* = 0.12), chicken meat (*P* = 0.19), and raw goat milk (*P* = 0.04), and low frequency (up to 10 times a year) of eating out of home (*P* = 0.01). Other behavioral characteristics including raising dogs, soil contact, degree of meat cooking, or eating unwashed raw fruits and vegetables were not associated with *Toxocara* seropositivity in bivariate analysis. Multivariate analysis (Table 4) of the 7 characteristics with a *P* value equal to or less than 0.20 obtained by bivariate analysis showed that a low frequency of eating out of home was positively associated with *Toxocara* exposure (OR  = 26; 95% CI: 2–363; *P*<0.05). In contrast, consumption of chicken meat was negatively associated with *Toxocara* exposure (OR  = 0.03; 95% CI: 0.003–0.59; *P*<0.05). No further behavioral characteristics of waste pickers associated with *Toxocara* exposure were found.

**Table 3:** Seroprevalence (%) of toxocaral infection in waste pickers relative to bivariate analysis of food consumption, feeding habits and other behavioural characteristics.

Characteristic	No. of subjects tested^a^	Prevalence	Odds ratio	95% Confidence interval	*P* value
Cats at home					
+	46	17.4	2.1	0.51–9.17	0.24
−	44	9.1	1.0		
Cats in the neighborhood					
+	63	17.5	5.5	0.67–120.06	0.07
−	27	3.7	1.0		
Cleaning cat excrement					
+	25	12	0.8	0.16–3.84	0.55
−	64	14.1	1.0		
Raising animals^b^					
+	36	13.9	1.1	0.27–4.28	0.56
−	54	13	1.0		
Dogs at home					
+	15	6.7	0.4	0.02–3.62	0.36
−	75	14.7	1.0		
Traveled abroad					
+	4	50	8.4	0.73–100.32	0.07
−	85	10.6	1.0		
National trips					
+	27	11.1	0.8	0.16–3.96	0.55
−	62	12.9	1.0		
Consumption of:					
Pork					
+	74	10.8	0.4	0.08–1.72	0.13
−	16	25	1.0		
Beef					
+	80	11.3	0.3	0.05–1.76	0.12
−	10	30	1.0		
Mutton					
+	34	8.8	0.5	0.10–2.28	0.25
−	56	16.1	1.0		
Chicken					
+	72	11.1	0.4	0.10–2.03	0.19
−	18	22.2	1.0		
Turkey					
+	48	16.7	1.9	0.46–8.28	0.24
−	42	9.5	1.0		
Raw cow milk					
+	25	12	0.9	0.16–3.91	0.56
−	65	13.8	1.0		
Raw goat milk					
+	3	66.7	15.4	0.95–476.41	0.04
−	87	11.5	1.0		
Chorizo					
+	73	15.1	2.8	0.33–63.09	0.28
−	17	5.9	1.0		
Unwashed raw vegetables					
+	29	18	0.7	0.13–3.03	0.41
−	61	14.8	1.0		
Unwashed raw fruits					
+	37	10.8	0.7	0.16–2.80	0.39
−	53	15.1	1.0		
Untreated water					
+	64	12.5	0.8	0.19–3.49	0.47
−	26	15.4	1.0		
Degree of meat cooking					
Undercooked	5	20	1.8	0.03–20.93	0.49
Well done	83	12	1.0		
Frequency of eating out of home					
Never	9	22.2	4.1	0.42–34.94	0.16
1–10 times a year	20	30	6.1	1.28–30.73	0.01
>10 times a year	61	6.6	1.0		
Soil contact					
+	36	16.7	1.6	0.40–6.18	0.33
−	53	11.3	1.0		
Floor at home					
Ceramic or wood	3	0	0.0	0.00–16.80	0.62
Concrete	39	15.4	1.0		
Soil	48	12.5	0.8	0.20–3.09	0.69

a Subjects with available data.

b Raising of any kind of animals.

+ =  Yes; − =  No.

**Table 4:** Results of multivariate analysis.

Variable	*P* value	Odds ratio	95% Confidence interval
Cats in the neighborhood	0.16	5.94	0.47–73.71
Traveled abroad	0.23	8.14	0.25–260.81
Pork consumption	0.33	0.25	0.01–3.97
Beef consumption	0.84	0.78	0.06–9.47
Chicken consumption	0.02	0.03	0.003–0.59
Raw goat milk consumption	0.05	46.54	1.00–2160.38
Low frequency of eating out of home	0.01	25.52	1.79–362.93

## Discussion

In this study, a significantly higher seroprevalence of anti-*Toxocara* IgG antibodies in waste pickers than in age- and gender-matched controls was found. Results clearly indicate that waste pickers represent a risk group for *Toxocara* infection. The 13% seroprevalence found in the present study indicates that *Toxocara* exposure is common among waste pickers; however, the lack of *Toxocara* seroprevalence studies in waste pickers does not allow comparing the prevalence of *Toxocara* seropositivity found with those in other waste pickers. However, in an international context, the seroprevalence in waste pickers is lower than those reported in some South American countries. *Toxocara* seroprevalences ranging from 21% to 39% have been reported in rural and urban populations in Bolivia [12], Brazil [13], Argentina [14]–[16], and Peru [17], [18]. In contrast, the seroprevalence in waste pickers is higher than the *Toxocara* seroprevalences less than 5% reported in Canada [19], [20]. The seroprevalence in waste pickers is comparable with the 14% seroprevalence reported in the USA [21]. The seroprevalence in waste pickers is also higher than those (2%–6%) reported in populations in Denmark [22], Korea [23], Spain [24], and India [25], and lower to the high (81%) *Toxocara* seroprevalence reported in Nepalese people [26], and to the 30% seroprevalence found in adults in Nigeria [27].

Concerning demographic characteristics, the *Toxocara* seroprevalence found in the present study was not significantly associated with any of the socio-demographic characteristics of the waste pickers. It has been reported that the prevalence of toxocariasis decreases with age [16]; however, *Toxocara* seroprevalence is waste pickers was not significantly influenced by age. This finding might suggest frequent *Toxocara* exposure in waste pickers. Toxocariasis has been associated with low educational level [28]. The lack of association of toxocariasis and educational level in waste pickers must be interpreted with care, since the great majority of waste pickers had a low educational level and a comparison with waste pickers with much higher educational level was not possible.

With respect to work characteristics, no significant associations of *Toxocara* seropositivity and work characteristics were found. The lack of association of *Toxocara* seropositivity with the years of working suggests that *Toxocara* exposure might occur early during the waste picking activity.

Of the clinical data, results indicate that *Toxocara* infection is impacting the health of waste pickers. Firstly, the prevalence of *Toxocara* seropositivity was significantly (*P*<0.05) higher in waste pickers suffering from gastritis than those without such clinical feature. To my knowledge, there is not previous report of an association of *Toxocara* infection and gastritis. Further research on the role of *Toxocara* infection in gastritis is needed. Secondly, seroprevalence of *Toxocara* infection was higher (borderline significance: *P* = 0.05) in waste pickers with visual impairment than those without this clinical feature. *Toxocara* infection is a known cause of eye disease [8], and further research on ocular toxocariasis in waste pickers is needed. Thirdly, waste pickers with reflex impairment had a significantly higher prevalence of *Toxocara* seropositivity that those without this clinical feature. To my knowledge there is not previous report of an association of *Toxocara* seropositivity and reflex impairment. Infection with *Toxocara* may lead to neurological involvement [29]; therefore, reflex impairment might be a manifestation of toxocariasis of the nervous system. Further research to confirm or challenge the association of *Toxocara* infection and reflex impairment is needed.

Concerning behavioral characteristics, multivariate analysis showed that *Toxocara* seropositivity was associated with a low frequency of eating out of home. This result suggests that infection with *Toxocara* was not acquired in restaurants or fast food outlets. Instead, it is possible that *Toxocara* exposure in waste pickers might have occurred at the waste transfer unit or at home. In addition, multivariate analysis showed that *Toxocara* seropositivity was negatively associated with chicken meat consumption. This finding suggests that chicken meat did not play a major role in *Toxocara* exposure in waste pickers.

*Toxocara* seropositivity has been associated with dog ownership [21], [28]. However, in the present study no association between *Toxocara* seropositivity and dog or cat ownership was found. This finding agrees with those found in other studies [27], [30]. In the present study, an association of *Toxocara* exposure and dog contact cannot be excluded because there were plenty dogs in the waste transfer unit and, therefore, waste pickers had contact with dogs at work. Contact with soil has been also found associated with toxocariasis [31]. However, in the present study such association was not found.

The conclusions of the present study are: 1) Waste pickers represent a risk group of *Toxocara* infection; 2) *Toxocara* is impacting the health of waste pickers. This is the first report of *Toxocara* infection in waste pickers and of associations of gastritis and reflex impairment with *Toxocara* seropositivity. Results warrant for further research.

## References

[1] Pelloux H, Faure O (2004) Toxocariasis in adults. Rev Med Interne 25: 201–206. 15049281 10.1016/s0248-8663(03)00258-3

[2] Rubinsky-Elefant G, Hirata CE, Yamamoto JH, Ferreira MU (2010) Human toxocariasis: diagnosis, worldwide seroprevalences and clinical expression of the systemic and ocular forms. Ann Trop Med Parasitol 104: 3–23. 20149289 10.1179/136485910X12607012373957

[3] Overgaauw PA (1997) Aspects of *Toxocara* epidemiology: human toxocarosis. Crit Rev Microbiol 23: 215–231. 9347221 10.3109/10408419709115137

[4] Despommier D (2003) Toxocariasis: clinical aspects, epidemiology, medical ecology, and molecular aspects. Clin Microbiol Rev 16: 265–272. 12692098 10.1128/CMR.16.2.265-272.2003 PMC153144

[5] Guillot J, Bouree P (2007) Zoonotic worms from carnivorous pets: risk assessment and prevention. Bull Acad Natl Med 191: 67–78. 17645108

[6] Magnaval JF, Glickman LT, Dorchies P, Morassin B (2001) Highlights of human toxocariasis. Korean J Parasitol 39: 1–11. 11301585 10.3347/kjp.2001.39.1.1 PMC2721060

[7] Sariego I, Kanobana K, Rojas L, Speybroeck N, Polman K, et al (2012) Toxocariasis in Cuba: a literature review. PLoS Negl Trop Dis 6: e1382. 22389726 10.1371/journal.pntd.0001382 PMC3289590

[8] Woodhall D, Starr MC, Montgomery SP, Jones JL, Lum F, et al.. (2012) Ocular Toxocariasis: Epidemiologic, Anatomic, and Therapeutic Variations Based on a Survey of Ophthalmic Subspecialists. Ophthalmology: (in press). 10.1016/j.ophtha.2011.12.013 22336630

[9] Smith H, Holland C, Taylor M, Magnaval JF, Schantz P, et al (2009) How common is human toxocariasis? Towards standardizing our knowledge. Trends Parasitol 25: 182–188. 19269251 10.1016/j.pt.2009.01.006

[10] Alvarado-Esquivel C, Liesenfeld O, Márquez-Conde JA, Cisneros-Camacho A, Estrada-Martínez S, et al (2008) Seroepidemiology of infection with *Toxoplasma gondii* in waste pickers and waste workers in Durango, Mexico. Zoonoses Public Health 55: 306–12. 18489540 10.1111/j.1863-2378.2008.01133.x

[11] Alvarado-Esquivel C, Estrada-Martínez S, Pizarro-Villalobos H, Arce-Quiñones M, Liesenfeld O, et al (2011) Seroepidemiology of *Toxoplasma gondii* infection in general population in a Northern Mexican city. J Parasitol 97: 40–3. 21348604 10.1645/GE-2612.1

[12] Cancrini G, Bartoloni A, Zaffaroni E, Guglielmetti P, Gamboa H, et al (1998) Seroprevalence of *Toxocara* canis-IgG antibodies in two rural Bolivian communities. Parassitologia 40: 473–475. 10645561

[13] Rubinsky-Elefant G, da Silva-Nunes M, Malafronte RS, Muniz PT, Ferreira MU (2008) Human toxocariasis in rural Brazilian Amazonia: seroprevalence, risk factors, and spatial distribution. Am J Trop Med Hyg 79: 93–98. 18606770

[14] Radman NE, Archelli SM, Fonrouge RD, del V Guardis M, Linzitto OR (2000) Human toxocarosis. Its seroprevalence in the city of La Plata. Mem Inst Oswaldo Cruz 95: 281–285. 10800184 10.1590/s0074-02762000000300001

[15] Chiodo P, Basualdo J, Ciarmela L, Pezzani B, Apezteguía M, et al (2006) Related factors to human toxocariasis in a rural community of Argentina. Mem Inst Oswaldo Cruz 101: 397–400. 16951810 10.1590/s0074-02762006000400009

[16] Fillaux J, Santillan G, Magnaval JF, Jensen O, Larrieu E, et al (2007) Epidemiology of toxocariasis in a steppe environment: the Patagonia study. Am J Trop Med Hyg 76: 1144–1147. 17556626

[17] Roldán WH, Cavero YA, Espinoza YA, Jiménez S, Gutiérrez CA (2010) Human toxocariasis: a seroepidemiological survey in the Amazonian city of Yurimaguas, Peru. Rev Inst Med Trop Sao Paulo 52: 37–42. 20305953 10.1590/s0036-46652010000100006

[18] Espinoza YA, Huapaya PE, Roldán WH, Jiménez S, Abanto EP, et al (2010) Seroprevalence of human toxocariasis in Andean communities from the Northeast of Lima, Peru. Rev Inst Med Trop Sao Paulo 52: 31–36. 20305952 10.1590/s0036-46652010000100005

[19] Sampasa-Kanyinga H, Lévesque B, Anassour-Laouan-Sidi E, Côté S, Serhir B, et al (2012) Zoonotic infections in native communities of James Bay, Canada. Vector Borne Zoonotic Dis 12: 473–481. 22217180 10.1089/vbz.2011.0739

[20] Messier V, Lévesque B, Proulx JF, Rochette L, Serhir B, et al (2012) Seroprevalence of seven zoonotic infections in Nunavik, Quebec (Canada). Zoonoses Public Health 59: 107–117. 21824376 10.1111/j.1863-2378.2011.01424.x

[21] Won KY, Kruszon-Moran D, Schantz PM, Jones JL (2008) National seroprevalence and risk factors for Zoonotic *Toxocara* spp. infection. Am J Trop Med Hyg 79: 552–557. 18840743

[22] Stensvold CR, Skov J, Møller LN, Jensen PM, Kapel CM, et al (2009) Seroprevalence of human toxocariasis in Denmark. Clin Vaccine Immunol 16: 1372–1373. 19641098 10.1128/CVI.00234-09 PMC2745020

[23] Park HY, Lee SU, Huh S, Kong Y, Magnaval JF (2002) A seroepidemiological survey for toxocariasis in apparently healthy residents in Gangwon-do, Korea. Korean J Parasitol 40: 113–117. 12325440 10.3347/kjp.2002.40.3.113 PMC2721037

[24] Jimenez JF, Valladares B, Fernandez-Palacios JM, de Armas F, del Castillo A (1997) A serologic study of human toxocariasis in the Canary Islands (Spain): environmental influences. Am J Trop Med Hyg 56: 113–115. 9063372 10.4269/ajtmh.1997.56.113

[25] Malla N, Aggarwal AK, Mahajan RC (2002) A serological study of human toxocariasis in north India. Natl Med J India 15: 145–147. 12186327

[26] Rai SK, Uga S, Ono K, Nakanishi M, Shrestha HG, et al (1996) Seroepidemiological study of *Toxocara* infection in Nepal. Southeast Asian J Trop Med Public Health 27: 286–290. 9279991

[27] Ajayi OO, Duhlinska DD, Agwale SM, Njoku M (2000) Frequency of human toxocariasis in Jos, Plateau State, Nigeria. Mem Inst Oswaldo Cruz 95: 147–149. 10733730 10.1590/S0074-02762000000200002

[28] Prestes-Carneiro LE, Souza DH, Moreno GC, Troiani C, Santarém V, et al (2009) Toxocariasis/cysticercosis seroprevalence in a long-term rural settlement, São Paulo, Brazil. Parasitology 136: 681–689. 19366477 10.1017/S0031182009005769

[29] Finsterer J, Auer H (2007) Neurotoxocarosis. Rev Inst Med Trop Sao Paulo 49: 279–287. 18026633 10.1590/s0036-46652007000500002

[30] Demirci M, Kaya S, Cetin E, Arıdoğan B, Onal S, et al (2010) Seroepidemiological investigation of toxocariasis in the isparta region of Turkey. Iran J Parasitol 5: 52–59. 22347244 PMC3279832

[31] El-Shazly AM, Abdel Baset SM, Kamal A, Mohammed KA, Sakrs TI, et al (2009) Seroprevalence of human toxocariasis (visceral larva migrans). J Egypt Soc Parasitol 39: 731–744. 20120741

